# Why Do Parents with Toddlers Store Poisonous Products Safely?

**DOI:** 10.1155/2010/702827

**Published:** 2010-07-01

**Authors:** Tinneke M. J. Beirens, Eduard F. van Beeck, Johannes Brug, Paul den Hertog, Hein Raat

**Affiliations:** ^1^Department of Public Health, Erasmus MC—University Medical Center Rotterdam, P.O. Box 2040, 3000 CA Rotterdam, The Netherlands; ^2^Consumer Safety Institute, 1070 AD Amsterdam, The Netherlands

## Abstract

Unintentional poisoning is a major cause of nonfatal injuries in children aged 0–24 months. Associations between self-reported habits on the child safe storage of medication and cleaning products and family, and psychosocial factors were assessed, using a model based on the Protection Motivation Theory. By identifying correlates of safety behavior in this manner, more insight in factors which influence this behavior is obtained. Health promotion activities in order to promote safety behavior should address these factors in order to increase the effectiveness of the health message. Data were gathered from a cross-sectional survey using self-administered questionnaires, mailed to a population sample of 2470 parents with toddlers. The results indicate that the promotion of safe storage of medication and cleaning products should address the family situation, personal cognitive factors as well as social factors. Interventions should particularly focus on parents' self-efficacy of storing poisonous products in a child safe manner and on the vulnerability of their child in their home concerning an unintentional poisoning incident.

## 1. Introduction

Unintentional poisonings, which mostly occur at home, are a major cause of nonfatal injuries in children aged 0–24 months [1]. In 2003, 49.6% of all the reported poisonings exposures in the USA occurred among preschool children [2]. The annual incidence of poisoning in Dutch children aged 1-2 years is 3 per 1000 children, which is much higher compared to other age groups, for example, one-year-olds have a six-fold risk of poisoning compared to four-year-olds [3]. Most cases of poisoning in children aged 1-2 years occur due to unsafe storage of medicines and cleaning products (e.g., detergents, chloride, and other cleaners) [3]. Unintentional poisonings in this age group can be largely prevented by taking preventive action. Examples of preventive action could include, according to guidelines, the use of child-resistant packaging, child-safe storage of potentially poisonous products, and extra attention and supervision when the possible hazardous products are in use [1, 4–8]. 

Efforts should be made to promote poisoning preventive actions, including child-safe storage of potentially poisonous products (e.g., placed above adult eye level, or in a locked cabinet) [1, 4–8]. Previous research showed a large variation in characteristics between parents who do and do not store medications or cleaning products child safe [9]. In order to develop effective intervention strategies to improve parental safety behavior, more insight into the underlying psychosocial mechanisms and potential important and modifiable mediators is needed.

Health promoting behaviors are influenced by a complex, interrelated set of so-called “mediators” or “determinants” of behaviors, which include various cognitions and environmental factors. By using behavioral theories, like the Protection Motivation Theory, one can identify the important determinants of a certain behavior. With this knowledge, one can go beyond basic unchangeable risk factors (e.g., gender, socioeconomic status) in order to explain why and how people change their behavior [10, 11]. Until now, use of behavioral theories and models in unintentional injury prevention research is only marginally represented in the literature [12]. 

The Protection Motivation Theory (PMT) is a framework particularly suited for interventions of protective, precautionary behaviors. According to the PMT, the probability of health protective behavior or an “adaptive response”—in this case (child) safe storage—is increased by four beliefs: (1) the threat is perceived as severe (severity), and (2) as of high personal relevance (vulnerability); (3) the adaptive response is perceived as effective for warding off the threat (response efficacy), and (4) the personal abilities and self-confidence to engage in the adaptive response is perceived as high (self-efficacy). According to the PMT, the probability of an adaptive response is decreased by the perceived rewards of a maladaptive response, for example, not using safety locks, and the perceived costs or barriers of the adaptive response (advantages and disadvantages of safe behavior) [13, 14]. 

We adopted this model for the present study to assess the influence of personal cognitions and attitudes on parental safety behavior and additionally included three social influence factors. 

 In the present study, we assessed the psychosocial correlates of parental safety behaviors concerning unintentional poisoning from medicines and cleaning products, among parents of toddlers aged 11–18 months in order to determine the most important changeable factors associated with safe storage of medications and cleaning products. A model based on the Protection Motivation Theory with the inclusion of additional social variables was applied.

## 2. Methods

### 2.1. Participants and Recruitment

In 2004 all parents (*n* = 2470) with at least one child aged 11–18 months who were part of an opportunity sample of six preventive youth healthcare providers in the Netherlands, both urban and rural, were invited by their preventive healthcare centers to complete a mailed questionnaire. The parents received a pre-survey letter from their healthcare provider informing them about the research and the survey was mailed to them by post. These six centers were chosen because of their ongoing collaboration with the Erasmus University Medical Center in Rotterdam. The preventive youth health care providers working in the centers have an average reach of parents of children in the age of 0–4 years old of 98%, indicating that all parents in the participating areas which fitted the selection criteria were invited to participate in the study. The parents were informed that the study was about home safety issues aiming to improve the safety information provided by preventive youth healthcare providers. Up to two reminders were sent by mail (one after 10 days and the other after 21 days), no financial incentives were offered, parents were assured of confidentiality, and the results were processed anonymously. One parent was asked to respond for each family to avoid dependent data. The Medical Ethics Committee of Erasmus MC approved the study.

### 2.2. Questionnaire

The questionnaire was developed and pilot tested among 25 parents, and refined based on interviews with these parents. These parents were requited through one of the participating centers. The questionnaire, with 160 questions, addressed child safe storage behavior of medication and cleaning products, standard socio-demographic variables as well as potential correlates of safe storage behavior (measured in PMT constructs).

#### 2.2.1. Safety Behavior

Storage was measured by asking whether the respondents stored the various products, “On the floor” or “In a drawer/cupboard without a lock, lower than 1.5 meter”, “In a drawer/cupboard without a lock, higher than 1.5 meter”, or “In a drawer/cupboard with a lock or safety catches”. The first two answers were considered as unsafe storage, and the latter two as storing the products in a child safe manner.

#### 2.2.2. Potential Correlates of Safety Behavior

The used model is mainly based on the Protection Motivation Theory. We adopted this model for the present study to assess the influence of personal cognitions and attitudes on parental safety behavior. In addition, we included three social influence factors in the explanatory model in order to assess the influence of these additional constructs on this behavior. The following social influence factors were assessed: perceived social support (or pressure), subjective norm, and descriptive norm. Perceived social support can be considered as the direct perceived influence of significant others (e.g., by receiving mental support to perform the desired behavior). Subjective norm is the perceived expectations of significant others (e.g., does my partner expect me to store medicines out of reach?). Descriptive norm refers to an individual's perception of how much and how often others perform the behavior [15–17].

Potential correlates of storage of possibly poisonous products were measured within the domain of PMT factors (perceived vulnerability and severity of the potential accident, response efficacy of safety preventive behaviors, general self-efficacy to perform safety preventive behaviors, and perceived advantages and disadvantages of safe behavior), psychosocial factors (active encouragement from other parents (social support), subjective norm, and descriptive norm), and demographic variables. The demographic variables included in this study were chosen based on earlier studies indicating the influence of these variables on safety behavior [9, 18] (i.e., age/walking ability of the child, number of children in the family, ethnicity/employment status/educational level of the mother and educational level of the father).

#### 2.2.3. PMT Constructs

Perceived vulnerability was measured by asking respondents their perception of the risk of their child unintentionally swallowing medicines or cleaning products (−2 = low risk; +2 = high risk). Perceived severity was measured with one item asking how seriously they perceived the consequences of this event (−2 not serious; +2 very serious). Response efficacy was measured by asking parents if they thought that storing medications and cleaning products out of reach of children could help to prevent possible accidents (−2 = not  very  helpful; +2 = very  helpful). 

Self-efficacy was assessed using items, which referred to the respondents' perception of their ability to store medications and cleaning products out of reach of children (−2 = very  difficult; +2 = very  easy). Perceived advantages of the safe behavior were assessed and measured with two questions (Spearman ranging from 0.70 to 0.77). Perceived disadvantages of the safe behavior were also assessed on a two-sided five-point scale and measured with two questions (Spearman ranging from 0.63 to 0.67).

All items to assess PMT and other psychosocial constructs were measured on bipolar five-point scales. For constructs that were assessed with multiple items, the mean score was calculated after sufficient internal consistency was established.

#### 2.2.4. Social Influence

Social support was measured by asking respondents if they received support from significant others to store medications and cleaning products out of reach of children (−2 = no support; +2 = many support). Subjective norm was assessed by asking if they perceived that their significant others thought storing medication and cleaning products out of reach of children is necessary, ranging from “certainly not” (−2) to “certainly yes” (+2). Descriptive norm was measured by asking respondents to assess how many people they perceived in their direct social environment to store medications and cleaning products out of reach of children in the same age category as their children (−2 = no body; +2 = every body).

#### 2.2.5. Demographics

Employment status of the parents was defined as employed when they had either a part-time or fulltime job. The educational level of the father and mother was divided into low and high (intermediate secondary education or less versus at least higher secondary education). Walking ability of the child was measured by asking whether the child could “walk independently, at least 2-3 steps”.

#### 2.2.6. Analyses

Categorical data were described using frequencies and percentages.

Differences in the proportions and means of all potential correlates in the model were tested by chi-square for the dichotomous demographic variables and Mann-Whitney *U* test for the PMT and social factors. To determine significant correlates of safe storage of medicines and cleaning products, multiple hierarchical logistic regression analyses were performed, with safe behavior as the dependent variable (No/Yes) and the various factors (demographic, PMT, and social) as independent variables. Two sets of multiple logistic regression analyses were conducted for safe storage of medicines and cleaning products, respectively. In both models, demographic variables were entered as a first block, since these variables were considered to be the more distal, nonmodifiable potential correlates. Subsequently blocks including the PMT (block 2) and social factors (block 3) were entered in the model. Explained variance was calculated with Nagelkerke *R* ^2^. Effect sizes were used as indicator of the explanatory value of the model [19]. All statistical analyses were performed using SPSS, Version 11.0.

## 3. Results

### 3.1. Participant Characteristics

Of the 2470 mailings to parents, the response rate was 70.1%. Nine questionnaires (0.5%) were excluded from the analyses because they had been incorrectly completed (*n* = 4) or because the questionnaire was not completed for the selected child but for an older sibling (*n* = 5); thus, 1722 questionnaires were used in the analyses. The mean age of the respondents (parent or guardian, no grandparents participated) was 32.4 years (range 16–60; SD 4.5); 90.1% were mothers. In this study, 97.5% of the families included two parents; 43.0% had one child (the child selected for the study). The age of the children ranged from 11 to 18 months (mean 13.5; SD 1.4); 47.0% were girls (Table 1).

### 3.2. Safe Storage of Medications and Cleaning Products

Medications and cleaning products were reported to be stored in a child-safe manner by, respectively, 74.4% (*n* = 1282) and 60.5% (*n* = 1042) of the respondents.

### 3.3. Differences between Safe and Unsafe Storage

Respondents who stored their medications or their cleaning products in a child-safe manner had a significantly lower perceived vulnerability, lower perceived disadvantages, higher perceived severity, self-efficacy and advantages of the safe behavior, and more positive social influences (Table 2). Respondents who stored their cleaning products in a child-safe manner also had a higher perceived response efficacy (Table 2).

### 3.4. Multiple Correlates of Safe Storage of Medications and Cleaning Products

The results of the multiple logistic regression analyses are shown in Table 3. Adding each block resulted in a significant increasing percentage of explained variance.

### 3.5. Safe Storage of Medication

In the first step, the number of children in the home was a significant variable, but this explained only 8% (Nagelkerke *R* ^2^) of the variance in the safe storage behavior. More than one child living in the home increased the likelihood that medication was stored in a child-safe manner. In the second step when PMT factors were entered, perceived vulnerability, self-efficacy, and disadvantages of the safe behavior were significantly associated with safe storage of medication and the explained variance increased to 22%. In the third step, when social factors were included, both social support and descriptive norm proved to be additional significant correlates and the explained variance increased to 24% indicating a medium effect size [19].

### 3.6. Safe Storage of Cleaning Products

In the first step, the number of children in the family was a significant variable but explained only 6% of the variance in safe storage of cleaning products. More than one child living in the home increased the likelihood that cleaning products were stored in a child safe manner. In the second step, perceived vulnerability, self-efficacy, and disadvantage of the safe behavior were also significantly associated with safe storage of cleaning products and together explained 32% of the variance. In the third step, social factors were included and these explained 33% of the variance in safe storage of cleaning products, indicating a large effect size. In addition, descriptive norm proved to be significantly correlated to safe storage of cleaning products.

## 4. Discussion

This study shows that perceived vulnerability, self-efficacy, perceived disadvantages of safe behavior, descriptive norm, and number of children in the family were significantly associated with safe storage of the studied potentially poisonous substances in households with toddlers. From our study, it can be concluded that the PMT model is applicable to predict the safe storage of possible poisons, even more so for storage of cleaning products than for medication. 

The associations of some of the separate psychosocial correlates included in our study were similar to the results in previous studies. For example, earlier studies showed differences in parents who do and do not take injury preventive behaviors in their perceptions of the vulnerability to an injury [11, 20], beliefs about the response efficacy of taking preventive measures [11], and perceived social norms [20, 21]. Furthermore, our results concerning the explained variance in safe storage (24–33%) are in line with Morrongiello & Kiriakou (2004) who were able to explain 32% of the variance in safety behavior related to prevention of poisonings in general [15]. 

The response rate was high, but we do not know whether families who refused to participate differed in demographic characteristics. However, based on findings of Kendrick et al. (2001), it is unlikely that the children of nonresponders differed from the responders in this study [22]. Furthermore, the demographic characteristics of the participants in our study (age, employment status, and educational level) reflected those of the general Dutch population and compare well with the distribution of these characteristics in a previous Dutch random sample of parents with preschool children [23, 24]. The results might be different in other locations but do seem to be representative for the Dutch situation, and comparable regions. 

Some limitations of this study need to be addressed. First, because our study relied on self-report of medication and cleaning products storage by parents, misclassification might have occurred, for example, parents might have given socially desirable answers (overstating safe storage and supervision of the child when products were stored unsafely) or might not have been fully aware of the identity of all poisonous products in their home [20, 25–27]. This might result in an underestimation of households with unsafe product storage, and bias in the assessment of significant correlates.

We were only able to assess the storage practices of parents related to potential poisonous substances (e.g., no information was available on child supervision during use of poisonous products). Additional data on the use of possible hazardous products might indicate a higher exposure to possible poisonings among children than found in this study and would have provided more insight into parents' injury preventive behavior concerning poisoning. On the other hand, this would have led to a more complex outcome measure and an increased probability of misclassification.

The parents who do not store their medications and cleaning products in a child-safe manner perceived their child to be more vulnerable to possible unintentional poisoning than parents who do store their products in a safe manner. Which is in practice a justified feeling of the parents, in their home these possible poisonous products are not stored safe, thus it is true that their child is probably more vulnerable to a poisoning injury. Furthermore, the parents who do not store the products in a child-safe manner estimate the severity of a possible poisoning as being lower than parents who do store their products in a child-safe manner. This lower estimation of the severity of a possible poisoning may explain why a subgroup of parents does not store the medication and cleaning products in a child-safe manner.

The strong significant contribution of both social influence (descriptive norm) and self-efficacy in the prediction of protection behavior indicates that parents are influenced by what they (perceive to) observe in their environment and what they perceive they themselves can do. Our data also show differences in the mean perceived severity and response efficacy of safe storage of medications and cleaning products, respectively. This suggests that even within the different types of injuries, in this case poisoning, determinants of engaging in safety practices vary. This finding indicates that safety promoting messages should not be generalized to overall injury prevention message, but that poisoning prevention, fall prevention, and burn prevention should be approached in various ways according to the most important determinants of the behavior. Further research should be executed to reject or support these findings, with that we recommend including a broader age and products range in future studies to give a better insight in possible poisonings in homes with young children.

### 4.1. Implications for Prevention

To increase parents' safe storage behavior, insight into potentially important and changeable mediators is needed when developing effective strategies. The study findings yield some recommendations for developing programs to prevent unintentional poisonings due to unsafe storage. Our study indicates that the promotion of safe storage of medication and cleaning products should address the family situation, and personal cognitive factors as well as social factors. For example, interventions focusing on behavioral change concerning prevention of poisoning should be open about the disadvantages of the safe behavior and with that point out that when performing the safe behavior the disadvantages will diminish. Furthermore, interventions should focus particularly on parents' self-efficacy, for example, show how can one store poisonous products safely, and perceived vulnerability of their child concerning a possible poisoning event, for example, point out hazardous situations in the home related to poisoning.

This study shows the importance of the perceived vulnerability of the parents child, perceived severity of an injury occurring, and response efficacy of a safety measure in the reason why parents do or do not perform safety preventive behaviours concerning poisoning prevention. 

Health promotion activities stimulating safe storage of these poisonous products should incorporate these constructs in their safety message in order to increase the effectiveness of the message.

## Figures and Tables

**Table 1:** Characteristics of family, child, history of previous injury, and poisoning preventive behavior (*n* = 1722 respondents).

Socio-demographic characteristics, history of previous injury and poisoning preventive behavior	*n*	% (unless otherwise specified)
Family characteristics		
Mean age of respondent in years	32.4	SD 4.5 Range 16–60
Mother is respondent	1541	90.1
Education level of mother is low^1^	1037	61.1
Education level of father is low^1^	1046	62.5
Mother is not employed	521	31.1
Father is not employed	54	3.2
Mother is of non-Dutch ethnicity	101	5.9
Father is of non-Dutch ethnicity	94	5.5
Single parent	33	1.9
One child in family	736	43.0

Child characteristics		
Mean age of child in months	13.5	SD 1.4 Range 11–18
Boy	901	52.7
Child can crawl	1664	97.3
Child can walk independently	811	47.5
Lifetime prevalence of medically attended unintentional injury	123	7.3

Poisoning preventive behavior		
Medications stored safely	1282	74.4
Cleaning products stored safely	1042	60.5

^1^Low educational level: intermediate secondary education or less.

**Table 2:** Differences between safe and unsafe storage of medications and cleaning products.

	Medications stored safely (*n* = 1282)	Medications stored unsafely (*n* = 425)	Cleaning products stored safely (*n* = 1042)	Cleaning products stored unsafely (*n* = 654)
Demographic variables				
Age of child is 11 through 13 months	49.2%	55.8%*	49.0%	54.1%*
Child cannot walk	51.1%	55.1%	50.0%	55.5%*
One child in family	36.9%	60.0%***	36.1%	53.2%***
Non-Dutch mother	5.5%	6.6%	4.6%	7.6%**
Mother is unemployed	32.4%	23.1%***	31.7%	27.2%*
Mother had lower education^1^	62.9%	53.2%***	62.9%	56.1%**
Father had lower education^1^	62.7%	55.5%**	63.5%	56.9%**

	*Mean (SD)*	*Mean (SD)*	*Mean (SD)*	*Mean (SD)*

PMT constructs				
Vulnerability (−2, +2)	−1.54 (0.74)	−1.16 (0.91)***	−1.43 (0.79)	−0.79 (0.99)***
Severity (−2, +2)	1.62 (0.74)	1.52 (0.82)**	1.75 (0.56)	1.60 (0.75)***
Response efficacy (−2, +2)	1.35 (0.97)	1.35 (0.94)	1.54 (0.83)	1.45 (0.87)**
Self-efficacy (−2, +2)	1.58 (0.78)	0.93 (1.09)***	1.57 (0.75)	0.66 (1.11)***
Advantages of safe behavior (−2, +2)	1.91 (0.35)	1.79 (0.46)***	1.90 (0.34)	1.68 (0.55)***
Disadvantages of safe behavior (−2, +2)	−1.69 (0.60)	−1.24 (0.90)***	−1.63 (0.65)	−1.01 (0.98)***

Social factors				
Social support (−2, +2)	0.34 (1.45)	0.02 (1.41)***	0.66 (1.33)	0.40 (1.33)***
Subjective norm (−2, +2)	1.68 (0.68)	1.59 (0.70)***	1.60 (0.70)	1.42 (0.85)***
Descriptive norm (−2, +2)	1.15 (0.80)	0.84 (0.84)***	1.14 (0.81)	0.80 (0.80)***

^1^Low educational level: intermediate level of secondary education or less.

Differences in mean scores between the safe and unsafe storage conditions were evaluated by Chi-square test and by Mann-Whitney *U*-test.

*significant at the 0.05 level.

**significant at the 0.01 level.

***significant at the 0.001 level.

**Table 3:** Stepwise multiple logistic regression analyses with reported safe storage of medication and cleaning products as dependent variables and demographic (step 1), Protection Motivation Theory variables (step 2), and additional factors (step 3) as independent factors (*n* = 1722).

	Medication stored safe			Cleaning products stored safe		
	Model 1 OR (95% CI)	Model 2 OR (95% CI)	Model 3 OR (95% CI)	Model 1 OR (95% CI)	Model 2 OR (95% CI)	Model 3 OR (95% CI)
*Demographic variables*						

Age of child is 11 through 13 months	0.87 (0.67–1.12)	0.86 (0.65–1.13)	0.89 (0.67–1.17)	0.93 (0.74–1.16)	0.93 (0.72–1.20)	0.91 (0.70–1.17)
Child cannot walk	0.84 (0.65–1.08)	0.81 (0.62–1.07)	0.84 (0.64–1.11)	0.79 (0.63–0.99)*	0.90 (0.69–1.16)	0.91 (0.71–1.18)
One child in family	0.40 (0.31–0.50)***	0.37 (0.29–0.48)***	0.38 (0.29–0.49)***	0.49 (0.39–0.60)***	0.48 (0.37–0.61)***	0.46 (0.36–0.59)***
Non-Dutch mother	0.75 (0.44–1.27)	0.84 (0.48–1.48)	0.80 (0.45–1.40)	0.50 (0.31–0.80)**	0.51 (0.30–0.88)*	0.50 (0.29–0.87)*
Mother is unemployed	1.25 (0.95–1.64)	1.23 (0.92–1.65)	1.23 (0.92–1.65)	1.06 (0.84–1.34)	1.05 (0.80–1.37)	1.07 (0.81–1.40)
Mother had lower education^1^	1.26 (0.97–1.62)	1.17 (0.89–1.54)	1.16 (0.88–1.52)	1.16 (0.93–1.46)	0.95 (0.73–1.23)	0.94 (0.72–1.21)
Father had lower education^1^	1.22 (0.94–1.57)	1.11 (0.85–1.46)	1.09 (0.83–1.43)	1.30 (1.04–1.63)*	1.30 (1.01–1.68)*	1.30 (1.01–1.68)*

*PMT-constructs*						

Perceived vulnerability		0.78 (0.67–0.91)***	0.79 (0.67–0.92)**		0.63 (0.55–0.73)***	0.64 (0.55–0.74)***
Perceived severity		1.10 (0.93–1.28)	1.08 (0.92–1.28)		1.02(0.84–1.23)	1.03 (0.85–1.25)
Response efficacy		0.94 (0.81–1.08)	0.91 (0.79–1.05)		1.02 (0.87–1.19)	0.99 (0.84–1.16)
Self-efficacy		1.70 (1.43–2.03)***	1.67 (1.41–2.01)***		1.92 (1.64–2.25)***	1.86 (1.58–2.18)***
Advantages of safe behavior		0.93 (0.66–1.30)	0.92 (0.65–1.31)		1.15 (0.82–1.61)	1.08 (0.76–1.53)
Disadvantages of safe behavior		0.73 (0.57–0.92)**	0.76 (0.60–0.96)*		0.73 (0.60–0.88)***	0.74 (0.61–0.90)**

*Social factors*						

Social support			1.11 (1.01–1.22)*			1.01 (0.92–1.11)
Subjective norm			0.89 (0.73–1.09)			1.00 (0.84–1.20)
Descriptive norm			1.36 (1.15–1.61)***			1.33 (1.12–1.57)***

Nagelkerke *R* ^2^	0.079	0.220	0.237	0.062	0.325	0.334

^1^Low educational level: intermediate level of secondary education or less.

*Significant at the 0.05 level **Significant at the 0.01 level ***Significant at the 0.001 level.

## References

[1] Vincenten J (2004). *Priorities for child safety in the European Union: Agenda for Action*.

[2] Watson WA, Litovitz TL, Klein-Schwartz W (2004). 2003 annual report of the American Association of Poison Control Centers Toxic Exposure Surveillance System. *American Journal of Emergency Medicine*.

[3] Dutch Injury Surveillance System (LIS) (2003). *Database of 1999–2003*.

[4] Hoy JL, Day LM, Tibballs J, Ozanne-Smith J (1999). Unintentional poisoning hospitalisations among young children in Victoria. *Injury Prevention*.

[5] Woolf AD, Saperstein A, Forjuoh S (1992). Poisoning prevention knowledge and practices of parents after a childhood poisoning incident. *Pediatrics*.

[6] Azizi BH, Zulkifli HI, Kassim MS (1994). Circumstances surrounding accidental poisoning in children. *Medical Journal of Malaysia*.

[7] Petridou E, Kouri N, Polychronopoulou A, Siafas K, Stoikidou M, Trichopoulos D (1996). Risk factors for childhood poisoning: a case-control study in Greece. *Injury Prevention*.

[8] Rodgers GB (2002). The effectiveness of child-resistant packaging for aspirin. *Archives of Pediatrics and Adolescent Medicine*.

[9] Beirens TMJ, van Beeck EF, Dekker R, Brug J, Raat H (2006). Unsafe storage of poisons in homes with toddlers. *Accident Analysis and Prevention*.

[10] Brug J, Oenema A, Ferreira I (2005). Theory, evidence and intervention mapping to improve behavioral nutrition and physical activity interventions. *International Journal of Behavioral Nutrition and Physical Activity*.

[11] Wortel E, de Geus GH, Kok G (1995). Behavioral determinants of mothers’ safety measures to prevent injuries of pre-school children. *Scandinavian Journal of Psychology*.

[12] Trifiletti LB, Gielen AC, Sleet DA, Hopkins K (2005). Behavioral and social sciences theories and models: are they used in unintentional injury prevention research?. *Health Education Research*.

[13] Rogers RW, Prentice-Dunn S, Gochman DS (1997). Protection motivation theory. *Handbook of Health Behavior Research I: Personal and Social Determinants*.

[14] Rogers R, Cacioppo J, Petty R (1983). Cognitive and physiological processes in fear-based attitude change: a revised theory of protection motivation. *Social Psychophysiology: A Sourcebook*.

[15] Morrongiello BA, Kiriakou S (2004). Mothers’ home-safety practices for preventing six types of childhood injuries: what do they do, and why?. *Journal of Pediatric Psychology*.

[16] Sellström E, Bremberg S (1996). Perceived social norms as crucial determinants of mother’s injury- preventive behaviour. *Acta Paediatrica*.

[17] Ajzen I (1988). *Attitudes, Personality and Behaviour*.

[18] Cialdini RB, Kallgren CA, Reno RR, Zanna MP (1991). A focus theory of normative conduct: a theoretical refinement and re-evaluation of the role of norms in human behaviour. *Advances in Experimental Social Psychology*.

[19] Cohen J (1988). *Statistical Power Analysis for the Behavioral Sciences*.

[20] de Vries H, Dijkstra M, Kuhlman P (1988). Self-efficacy: the third factor besides attitude and subjective norm as a predictor of behavioural intentions. *Health Education Research*.

[21] Morrongiello BA, Ondejko L, Littlejohn A (2004). Understanding toddlers’ in-home injuries: I. Context, correlates, and determinants. *Journal of Pediatric Psychology*.

[22] Kendrick D, Hapgood R, Marsh P (2001). Do safety practices differ between responders and non-responders to a safety questionnaire?. *Injury Prevention*.

[23] Statline (2006). *Statistics Netherlands-Statline*.

[24] Raat H, Landgraf JM, Oostenbrink R, Moll HA, Essink-Bot M-L (2007). Reliability and validity of the Infant and Toddler Quality of Life Questionnaire (ITQOL) in a general population and respiratory disease sample. *Quality of Life Research*.

[25] Chen LH, Gielen AC, McDonald EM (2003). Validity of self reported home safety practices. *Injury Prevention*.

[26] Schwebel DC, Brezausek CM (2004). The role of fathers in toddlers’ unintentional injury risk. *Journal of Pediatric Psychology*.

[27] Watson M, Kendrick D, Coupland C (2003). Validation of a home safety questionnaire used in a randomised controlled trial. *Injury Prevention*.

